# Professional learning and development of postdoctoral scholars: a scoping review protocol

**DOI:** 10.1186/s13643-018-0892-5

**Published:** 2018-12-05

**Authors:** Lorelli Nowell, K. Alix Hayden, Carol Berenson, Natasha Kenny, Nancy Chick, Carolyn Emery

**Affiliations:** 1 Taylor Institute for Teaching and Learning, University of Calgary, 434 Collegiate Boulevard NW, Calgary, AB T2N 1N4 Canada; 2 Libraries and Cultural Resources, University of Calgary, Calgary, Canada; 3 Sport Injury Prevention Research Centre, Faculty of Kinesiology, University of Calgary, Calgary, Canada; 4 Department of Paediatrics, Cumming School of Medicine, University of Calgary, Calgary, Canada; 5 Department of Community Health Sciences, Cumming School of Medicine, University of Calgary, Calgary, Canada; 6 Alberta Children’s Hospital Research Institute, Cumming School of Medicine, University of Calgary, Calgary, Canada; 7 Hotchkiss Brain Institute, Cumming School of Medicine, University of Calgary, Calgary, Canada; 8 O’Brian Institute for Public Health, Cumming School of Medicine, University of Calgary, Calgary, Canada

**Keywords:** Scoping review, Postdoctoral scholar, Postdoctoral fellow, Professional development, Professional learning

## Abstract

**Background:**

A growing number of postdoctoral scholars are following diverse career paths that require broad skill sets to ensure success. Yet, most postdoctoral professional learning and development initiatives are intended for academic careers and seldom include professional skills needed to succeed in non-academic settings. Given that fewer than 20% of postdoctoral scholars will obtain tenure-track academic positions, there is a great need for postdoctoral scholars to prepare for a range of future careers. Creating professional learning and development strategies to address these concerns requires an understanding of current approaches, yet there is a distinct lack of literature exploring and synthesizing sources of evidence on the professional learning and development of postdoctoral scholars. The purpose of this scoping review is to examine, synthesize, and map the sources of evidence on professional learning and development pertaining to postdoctoral scholars.

**Methods:**

We will perform a scoping review to identify sources of evidence around professional learning and development of postdoctoral scholars. Our search strategy, limited to English language, will include searching relevant disciplinary and interdisciplinary databases with no limitation on date of publication. We will conduct forward and backward citation chasing of included articles. Gray literature will be searched in electronic databases and websites of national postdoctoral associations. Search strategies will be developed using controlled vocabulary and keyword terms related to postdoctoral scholars and professional development. Two reviewers will independently screen titles and abstracts for inclusion, and two reviewers will independently screen full text to determine final inclusion. These data will be summarized quantitatively (using a simple numerical count) and qualitatively using thematic analysis methods. Through this process, we will summarize the current state of evidence around professional development and learning of postdoctoral scholars and identify current gaps in the literature, as well as the research areas requiring systematic reviews and/or primary research.

**Discussion:**

Despite the growing numbers of postdoctoral scholars, there has been no synthesis of the sources of evidence of postdoctoral scholars’ professional learning and development. In reviewing a wide range of evidence and integrating it into a manageable and meaningful whole, this scoping review will be a critical first step in understanding the professional learning and development of postdoctoral scholars. Our results will help inform future research and the development of a framework for postdoctoral scholar’s professional learning and development.

**Electronic supplementary material:**

The online version of this article (10.1186/s13643-018-0892-5) contains supplementary material, which is available to authorized users.

## Background

Postdoctoral scholars hold doctoral degrees and are engaged in mentorship opportunities to develop their scientific independence, academic merit, and entrepreneurial research skills [1]. They are central to the advancement of scientific inquiry, improvement of teaching practices in higher education, studying relevant problems, addressing important societal issues, and informing future policy [2, 3]. Postdoctoral scholars are significant members of the research community, making important contributions to research productivity [4, 5], contributing to knowledge translation, and strengthening collaborative research networks [5].

Postdoctoral fellowships are traditionally short-term positions (1–5 years) intended to bridge the gap between PhD completion and a tenure-track faculty position [2, 3]. However, the increasing number of postdoctoral scholars has far outpaced universities’ needs for new academic faculty [2, 3, 6, 7] and fewer than 20% of current postdoctoral scholars are likely to obtain tenure-track positions [8]. As a result of this trend, today’s postdoctoral scholars are following more diverse career paths and increasingly pursuing careers in industry, government, and beyond, or leaving research altogether [7].

As increasing number of postdoctoral scholars pursue careers outside of academia, the need to develop a broad skill set to succeed in their various roles also increases. Worldwide, educators, business leaders, and politicians have developed lists and models of twenty-first century skills that professionals need both today and in the future [9, 10]. Recent studies from different fields indicates that the skills professionals need are not only field-specific skills but also include broad skills, such as social skills, organizing skills, skills for knowledge acquisition, and problem-solving skills [11–13]. Success in many careers also requires interpersonal communication, presentation, leadership, management, networking, and teaching skills [14]. Despite these findings, traditional emphasis has been placed on developing research skills and scientific knowledge, resulting in insufficient resources being dedicated to the broader professional learning and development of postdoctoral scholars.

We purposefully use the term “professional learning and development” within the context of our work to ensure a broad focus on the experience and continuous nature of professional learning and professional development, by engaged individuals capable of self-directed learning [15, 16]. Professional learning and development is situated, social and constructed, and is based on a complex relationship between individuals and their environment [16, 17]. It includes a range of formal and/or informal activities and interactions, as well as contextual learning and reflective practice that may increase postdoctoral scholars’ knowledge, skills, abilities, and growth while improving their performance in present or future positions [16, 18]. Professional learning and development experiences may range from formal structured initiatives (e.g., seminars, workshops, conferences, courses), to embedded professional and self-directed learning activities (e.g., co-teaching, mentorship, group discussions, communities of practice, professional meetings, reading groups), to informal everyday discussions and work-related practices with other researchers, educators, and scholars [16, 17, 19].

Postdoctoral scholars recognize a need for sufficient opportunities to prepare for the various roles and responsibilities of their diverse future positions [1, 20, 21]. Yet, most professional learning and development opportunities for postdoctoral scholars are intended to prepare them for academically focused research careers and rarely include professional skills needed to succeed more broadly in their academic careers or in non-academic settings [2, 3, 7]. Though much importance has been placed on developing professional learning and development programs for graduate student, far fewer programs occur for postdoctoral scholars [21–24]. There is a scarcity of literature exploring and synthesizing sources of evidence on the professional learning and development of postdoctoral scholars. Based on this background, our objective is to conduct a scoping review to examine and synthesize the sources of evidence on professional learning and development of postdoctoral scholars.

### Logic model

**Fig. 1:**
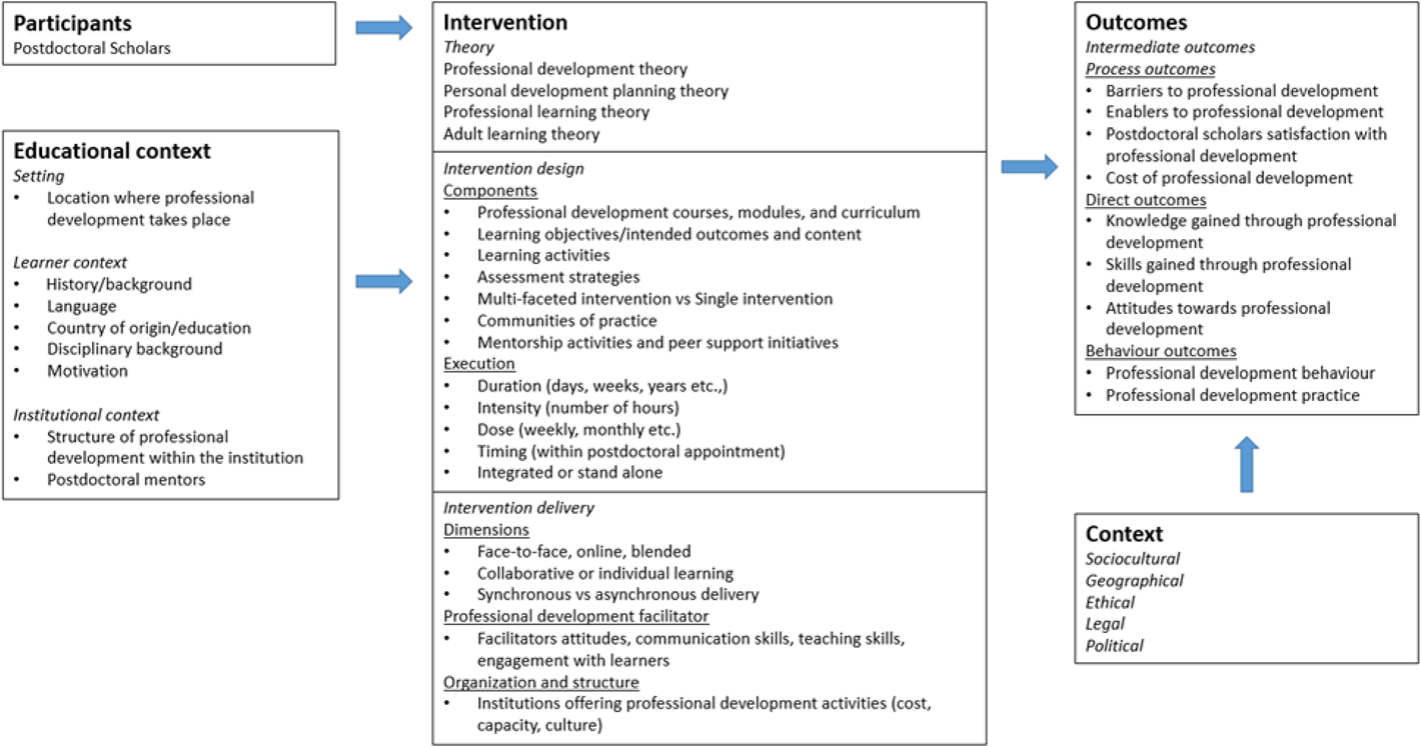
Logic model demonstrating assumed relationship between postdoctoral scholars and professional development

Logic models offer graphic depictions of important elements and relationships related to a specific topic [25]. When used in research synthesis, such as scoping reviews, logic models can facilitate collecting and integrating literature to better inform interpretations and aggregation of results [26]. Using a logic model helps conceptualize the complexity of professional learning and development by (1) depicting professional learning and development components and relationships between them, (2) explicating pathways between professional learning and development and multiple outcomes, and (3) displaying interactions between professional learning and development and the context in which it is implemented [25]. Our logic model (Fig. 1) provides a framework for identifying inclusion and exclusion criteria, guiding the search strategy, and examining differences in the literature and along dimensions of interest [25]. Furthermore, our logic model offers a transparent graphic representation of our assumptions which is useful to other researchers and stakeholders making our results more accessible to a broad range of decision-makers [27].

## Methods and analysis

Various systematic approaches are available for reviewing published literature. Scoping reviews are a rigorous and methodical approach to examining the extent, range, and nature of research activity in a particular field [28]. We believe a scoping review of published literature is the best method to map the global literature regarding professional learning and development of postdoctoral scholars. Scoping review methodology will guide us to broadly and comprehensively examine and systematically map sources of evidence on professional learning and development of postdoctoral scholars, determine feasibility of conducting subsequent systematic reviews, summarize and disseminate research findings to knowledge users, and identify gaps where further research is required [28, 29]. In designing the protocol for our scoping review, we drew upon Arskey and O’Malley’s [28] seminal work as well as more recent scoping review publications [29–32]. The review will include the following stages: (1) formulating the research questions; (2) identifying relevant literature; (3) study selection based on clear inclusion and exclusion criteria; (4) charting the data using standardized data extraction tables; (5) collating, summarizing, and reporting the results including an appraisal of the literature; and (6) consultation. In addition, this scoping review will include an evidence map offering a user-friendly format illustrating the evidence and gaps in evidence that exist for professional learning and development of postdoctoral scholars. To ensure the evidence map is useful to stakeholders, we will draw upon Bragge and colleagues framework for global evidence mapping [33] and more recent evidence map literature [34, 35]. A completed PRISMA-P (Preferred Reporting Items for Systematic Review and Meta-Analysis Protocols) checklist has been submitted as an additional file to this protocol (Additional file 1). This study has not been registered in PROSPERO as scoping reviews are not eligible for inclusion.

### Stage 1: Identifying the research questions

In scoping reviews, research questions should be broad with the focus on summarizing the breadth of literature [28]. Our overarching research question is as follows: What is known from existing sources of evidence about the professional learning and development of postdoctoral scholars? Our objectives are the following: first, to describe which professional learning and development opportunities are being reported worldwide; second, to explore how professional learning and development opportunities are being used by postdoctoral scholars; third, to categorize professional learning and development opportunities that are relevant to meeting the needs of postdoctoral scholars; and fourth, to identify optimal strategies for providing professional development to postdoctoral scholars.

### Stage 2: Identifying relevant literature

Scoping reviews aim to comprehensively address broad research questions; however, parameters are required to guide the search strategy. Together, our review team deliberated and decided upon the criteria for eligibility and databases to search and formulated a search strategy.

#### Eligibility criteria

We will include sources of evidence on professional learning and development of postdoctoral scholars including studies, theses/dissertations, and gray literature. For the purpose of this review, postdoctoral scholars are defined as scholars who hold doctoral degrees and are engaged in mentored research and/or scholarly training for the purpose of skills development [1]. Professional learning and development is defined as any activities and interactions that may increase postdoctoral scholars’ knowledge and skills; contribute to their personal, social, and emotional growth as scholars; and improve their performance in present or future roles. These experiences can range from formal structured formats (seminars, workshops, conferences, courses), to embedded professional development (co-teaching, mentorship, group discussions, communities of practice), to informal discussions with other researchers, educators, and scholars.

#### Information sources and search strategy

Together, the team recognized the need for the search to encompass as many disciplines as possible in order to exhaustively capture the literature related to postdoctoral scholars and professional development. Databases were identified by conducting a simple search using “postdoc*” as a keyword to determine whether the concept was represented in the database and to make an informed decision as to which databases to search. Disciplinary databases to be searched include Business Source Complete, Biosis Previews, CAB Abstracts, CINAHL, Communication Abstracts, Education Resources Complete, EMBASE, Environment Complete, ERIC, IEEE Xplore, MEDLINE, PsycINFO, MEDLINE, and SocIndex. Interdisciplinary databases to be searched include Academic Search Complete, Scopus, and Web of Science. All databases will be searched from database inception for English language publications. Gray literature will be searched for in the ProQuest Dissertations and Theses Global database, Trove (National Library of Australia theses/dissertations), Ethos (British Library theses/dissertations), and websites of national postdoctoral associations (Canadian Association of Postdoctoral Scholars, National Postdoctoral Association). The search strategy was developed by an information scientist (KAH) and focuses on two main concepts: postdoctoral and professional development. For each concept, subject headings and keywords will be generated. Keywords will be constant across all databases, and subject headings will be responsive to the controlled vocabulary of the database when available. Table 1 outlines the search strategy for Ovid MEDLINE which yielded 677 articles. This search strategy will be adapted for each database. We will conduct forward and backward citation chasing of included articles. Records will be exported to EndNote v8 to enable data management, removal of duplicates, and retrieving full texts.

**Table 1:** Provisional search strategy

Number	Searches	Results
1	postdoc*.mp.	1709
2	post-doc*.mp.	498
3	post-phd*.mp.	10
4	or/1-3	2198
5	exp Staff Development/	8947
6	exp Leadership/	37,479
7	exp Mentoring/	411
8	exp Mentors/	9818
9	exp Teaching/	80,713
10	(professional adj1 development).mp.	7742
11	(professional adj1 learning).mp.	278
12	(professional adj1 growth).mp.	832
13	(career adj1 development).mp.	1962
14	(career adj1 mentor*).mp.	105
15	(career adj1 goal*).mp.	438
16	(career adj1 preparation).mp.	60
17	(career adj1 navigat*).mp.	5
18	(capacity adj1 development).mp.	538
19	(postdoc* adj2 train*).mp.	292
20	(faculty adj1 development).mp.	2370
21	(collegial adj1 mentor*).mp.	3
22	(peer adj1 coach*).mp.	149
23	coaching.mp.	4592
24	mentor*.mp.	17,695
25	(faculty adj3 learning communit*).mp.	19
26	work life balance.mp.	932
27	lifelong learn*.mp.	1199
28	transformative learn*.mp.	196
29	(talent adj1 management).mp.	50
30	(communit* adj1 practice*).mp.	2271
31	leadership.mp.	54,444
32	(teaching adj1 development).mp.	93
33	(teaching adj1 skill*).mp.	1247
34	(academic adj1 skill*).mp.	659
35	(academic adj1 development).mp.	311
36	(skill* adj1 development).mp.	2319
37	(training adj1 program*).mp.	38,395
38	(talent adj1 development).mp.	162
39	(skill* adj1 train*).mp.	7007
40	(education* adj1 development).mp.	784
41	or/5-40	210,492
42	4 and 41	689
43	limit 42 to english language	677

Database(s): Ovid MEDLINE (R) Epub Ahead of Print, In-Process & Other Non-Indexed Citations, Ovid MEDLINE (R) Daily, and Ovid MEDLINE (R) 1946 to Present Search Strategy

### Stage 3: Study selection

The selection of literature will occur in two phases. The first phase will involve screening of titles and abstracts by two reviewers, independently using a structured screening form based on the identified eligibility criteria. To minimize the risk of bias, screening forms will be pilot tested by reviewers on a random selection of 100 titles and abstracts to ensure consistency and reliability. A kappa [36] of greater than 0.8 will be used to quantify inter-investigator agreement. Disagreements will be resolved to consensus through discussion and passed to a third investigator for final resolution if the issue cannot be resolved. Literature identified as potentially relevant will be passed to the next screening level.

In phase 2, two reviewers will independently review full-text versions of all potentially relevant literature. To minimize the risk of bias, both reviewers will be trained on the phase 2 screening form prior to beginning screening and the forms will be pilot tested by the reviewers on a random selection of 10 full texts to ensure consistency and reliability between the reviewers. A kappa [36] of greater than 0.8 will be used to quantify inter-investigator agreement, and disagreements will be resolved by discussion. Unresolved disagreements will be referred to a third investigator for review and resolution.

A PRISMA (Preferred Reporting Items for Systematic Reviews and Meta-Analyses) flow diagram [37] will be used to report final numbers once the review is completed. A unique identifier will be assigned to every publication retrieved during data collection to enable tracking of the article throughout the review process. Endnote v8 will be used to manage the results of the searches. The final search results will be exported into an excel workbook to facilitate the screening process with each reviewer documenting the inclusion/exclusion status for each article.

### Stage 4: Charting the data

A “descriptive analytical” method will be used to extract contextual information from each included article, involving the synthesis and interpretation of the data by sifting, charting, and sorting material according to key findings and themes [28]. The review team will collectively develop a data extraction form guided by our logic model to determine which variables or themes to extract. This will be an iterative process, and the form will be continually updated as new key findings emerge [29].

Two reviewers will independently read each included article. One reviewer will extract relevant data using a standardized data extraction tool, and another reviewer will verify the extracted data for consistency and accuracy. The data extracted will include year, authors, publication title, research question or study purpose, study design, context, participants, sample size, theoretical/conceptual framework, interventions, definitions of concepts, data collection methods, and relevant results. Additional categories may be identified through completion of the search and in consultation with the team members. Study quality is generally not conducted during a scoping review [28, 29]; however, we will include a summary of literature limitations identified in articles included in the review.

### Stage 5: Collating, summarizing, and reporting the results

The unique purpose of a scoping review is to aggregate the findings and present an overview of the current state of evidence on a topic. The data arising from our data collection process will be summarized quantitatively (using a simple numerical count identifying numbers of studies) and qualitatively using thematic analysis methods [29]. Study findings will be inductively coded [38], compared between studies, and grouped to generate initial descriptive themes. Analytical themes will be created by exploring how the descriptive themes are linked between the studies. In addition, we will include an evidence map to illustrate the evidence gaps that exist for professional learning and development of postdoctoral scholars. Through this process, we will be able to summarize and critically evaluate the current state of evidence around professional learning and development of postdoctoral scholars and identify current gaps in the literature and professional learning and development programming, as well as the research areas which require systematic reviews or primary research.

### Stage 6: Consultation

The consultation phase is optional in the seminal Arskey and O’Malley framework [28]; however, we have chosen to include this step in our review. Obtaining feedback from relevant stakeholders will help ensure our results are relevant to the target audience [39]. We are using an integrated knowledge translation [40] approach for this scoping review, and our multidisciplinary team includes knowledge users (postdoctoral program director, postdoctoral scholars, director of educational development), knowledge synthesis methodologists, an information scientist, and experienced researchers. Our team co-developed the review question and protocol and will continue to be involved throughout the entire process, including the development of a knowledge translation strategy to increase the uptake of the findings of the review. The anticipated goal of engaging these individuals is to accelerate spread and use of knowledge and to establish evidence-based decision-making regarding professional development supports for postdoctoral scholars.

## Discussion

As highly trained and experienced early career researchers, postdoctoral scholars play a key role in driving discovery and expanding knowledge [2, 3]. Although there is an increasing interest for postdoctoral scholars to engage in professional learning and development opportunities, to date, there has been no synthesis of evidence to fully understand postdoctoral professional learning and development and strategies implemented to support their growth. The results of this work will be of use to several target audiences, including postdoctoral scholars, institutional decision-makers, and researchers, provincially, nationally, and internationally who have postdoctoral programs.

The main goal of our knowledge translation plan is to disseminate the research findings in formats and through pathways most appropriate for the target audiences. Our approaches to knowledge dissemination will include webinars via the Educational Development Caucus (an affiliate of the Society for Teaching and Learning in Higher Education) and presentations at national and international educational development and higher education conferences (such as the International Society for the Scholarship of Teaching and Learning Conference, and the International Consortium for Educational Development). At the local level, highlights of learnings will be disseminated through our University newsletters, blogs, annual reports, and conferences. A world café [28–30] with a graphic facilitator will be held with postdocs to discuss and build upon key study findings.

The results from this scoping review will help inform efforts to refine existing or develop new approaches to promote professional learning and development amongst postdoctoral scholars. Furthermore, the results will guide future work towards developing professional learning and development evaluation frameworks for postdoctoral scholars. The creation of an evidence-based professional learning and development framework will help advance theoretical understanding of postdoctoral professional learning and development and enhance the transferability of findings to multiple postsecondary contexts.

## Additional file

Additional file 1: PRISMA-P 2015 Checklist. (DOCX 25 kb)

## References

[1] Nerad M, Cerny J (1999). Postdoctoral patterns, career advancement, and problems. Science.

[2] Mitchell J, Walker V, Annan R, Corkery T, Goel N, Harvey L, Kent D, Peters J, Vilches S (2013). The 2013 Canadian postdoc survey: painting a picture of Canadian postdoctoral scholars. Canadian Association of Postdoctoral Scholars and Mitacs.

[3] Jadavji N, Adi M, Corkery T, Inoue J, Van Benthem K: The 2016 Canadian national postdoctoral survey report. In*.*; 2016.

[4] Vogel G (1999). A day in the life of a topflight lab. Science.

[5] Clotfelter CT. American universities in a global market. Chicago: University of Chicago Press; 2010.

[6] McKenzie M (2007). Where are the scientists and engineers?.

[7] Fuhrmann CN, Halme DG, O’Sullivan PS, Lindstaedt B (2011). Improving graduate education to support a branching career pipeline: recommendations based on a survey of doctoral students in the basic biomedical sciences. CBE-Life Sciences Education.

[8] Edge J, Munro D (2015). Inside and outside the academy: valuing and preparing PhDs for careers.

[9] Fadel C, Bialik M, Trilling B (2015). Four-dimensional education: the competencies learners need to succeed, vol. null.

[10] Gordon J, Halasz G, Krawczyk M, Leney T, Michel A, Pepper D, Putkiewicz E, Wisniewski J: Key competences in Europe: opening doors for lifelong learners across the school curriculum and teacher education, vol. null; 2009.

[11] Arevalo J, Pitkänen S, Gritten D, Tahvanainen L: International Forest Review 2010, 12(3):200.

[12] Tynjälä P, Slotte V, Nieminen J, Lonka K, Olkinuora E, Tynjälä P, Välimaa J, Boulton-Lewis G: High Educ and Working life: collaborations, confrontations and challenges, vol. null; 2006.

[13] Virtanen A, Tynjälä P, Stenström ML, Billett S, Harteis C, Eteläpelto A: Emerging perspectives of workplace learning, vol. null; 2008.

[14] Smith S, Pedersen-Gallegos L, Riegle-Crumb C (2002). The training, careers, and work of Ph.D. physical scientists: not simply academic. Am J Phys.

[15] Caffarella RS, Zinn LF (1999). Professional development for faculty: a conceptual framework of barriers and supports. Innov High Educ.

[16] Webster-Wright A (2009). Reframing professional development through understanding authentic professional learning. Rev Educ Res.

[17] Knight P, Tait J, Yorke M (2006). The professional learning of teachers in higher education. Stud High Educ.

[18] Åkerlind G (2005). Postdoctoral researchers: roles, functions and career prospects. Higher Education Research & Development.

[19] Roxå T, Mårtensson K (2009). Significant conversations and significant networks – exploring the backstage of the teaching arena. Stud High Educ.

[20] Brownell SE, Tanner KD (2012). Barriers to faculty pedagogical change: lack of training, time, incentives, and…tensions with professional identity?. CBE-Life Sciences Education.

[21] Rybarczyk B, Lerea L, Lund PK, Whittington D, Dykstra L (2011). Postdoctoral training aligned with the academic professoriate. BioScience.

[22] Kenny N, Watsons G, Watton C (2014). Exploring the context of Canadian graduate student teaching certificates in university teaching. Can J High Educ.

[23] Rose M. Graduate student professional development: a survey with recommendations. Ottawa: Canadian Association for Graduate Studies; 2012.

[24] Scaffidi AK, Berman JE (2011). A positive postdoctoral experience is related to quality supervision and career mentoring, collaborations, networking and a nurturing research environment. High Educ.

[25] Anderson LM, Petticrew M, Rehfuess E, Armstrong R, Ueffing E, Baker P, Francis D, Tugwell P (2011). Using logic models to capture complexity in systematic reviews. Res Synth Methods.

[26] Joffe M, Mindell J (2006). Complex causal process diagrams for analyzing the health impacts of policy interventions. Am J Public Health.

[27] Rohwer A, Pfadenhauer L, Burns J, Brereton L, Gerhardus A, Booth A, Oortwijn W, Rehfuess E (2017). Series: clinical epidemiology in South Africa. Paper 3: logic models help make sense of complexity in systematic reviews and health technology assessments. J Clin Epidemiol.

[28] Arksey H, O’Malley L (2005). Scoping studies towards a methodological framework. Int J Soc Res Methodol.

[29] Levac D, Colquhoun H, O'Brien K. Scoping studies: advancing the methodology. Implement Sci. 2010;5(69). 10.1186/1748-5908-5-69 PMC2954944 20854677

[30] Colquhoun H, Levac D, O'Brien K, Straus S, Tricco A, Perrier L, Kastner M, Moher D (2014). Scoping reviews: time for clarity in definition, methods, and reporting. J Clin Epidemiol.

[31] Daudt HML, van Mossel C, Scott SJ (2013). Enhancing the scoping study methodology: a large, inter-professional team's experience with Arksey and O’Malley’s framework. BMC Med Res Methodol.

[32] Joanna Briggs Institute (2015). The Joanna Briggs Institute reviewers’ manual 2015: Methodology for JBI’s scoping review.

[33] Bragge P, Clavisi O, Turner T, Tavender E, Collie A, Gruen RL (2011). The global evidence mapping initiative: scoping research in broad topic areas. BMC Med Res Methodol.

[34] Miake-Lye IM, Hempel S, Shanman R, Shekelle PG (2016). What is an evidence map? A systematic review of published evidence maps and their definitions, methods and products. Systematic Reviews.

[35] Snilstveit B, Vojtkova M, Bhavsar A, Gaarder M (2013). Evidence gap maps—a tool for promoting evidence-informed policy and prioritizing future research.

[36] Landis J, Koch G (1977). The measurement of observer agreement for categorical data. Biometrics.

[37] Liberati A, Altman D, Tetzlaff J, Mulrow C, Gotzsche P, Ioannidis J, Clarke M, Devereaux P, Kleijnen J, Moher D (2009). The PRISMA statement for reporting systematic reviews and meta-analyses of studies that evaluate healthcare interventions: explanation and elaboration. BMJ.

[38] Harden A, Thomas J (2005). Methodological issues in combining diverse study types in systematic reviews. Int J Soc Res Methodol.

[39] Tricco AC, Lillie E, Zarin W, O’Brien K, Colquhoun H, Kastner M, Levac D, Ng C, Sharpe JP, Wilson K (2016). A scoping review on the conduct and reporting of scoping reviews. BMC Med Res Methodol.

[40] Graham ID, Logan J, Harrison MB, Straus SE, Tetroe J, Caswell W, Robinson N (2006). Lost in knowledge translation: time for a map?. J Contin Educ Health Prof.

